# Bioactivity and efficacy of a hyperimmune bovine colostrum product- Travelan, against shigellosis in a non-Human primate model (*Macaca mulatta*)

**DOI:** 10.1371/journal.pone.0294021

**Published:** 2023-12-13

**Authors:** Dilara Islam, Nattaya Ruamsap, Rawiwan Imerbsin, Patchariya Khanijou, Siriphan Gonwong, Matthew D. Wegner, Annette McVeigh, Frédéric M. Poly, John M. Crawford, Brett E. Swierczewski, Robert W. Kaminski, Renee M. Laird

**Affiliations:** 1 US Army Medical Directorate of the Armed Forces Research Institute of Medical Sciences (USAMD-AFRIMS), Bangkok, Thailand; 2 Henry M. Jackson Foundation for Military Medicine (HJF), Bethesda, Maryland, United States of America; 3 Naval Medical Research Command (NMRC), Silver Spring, Maryland, United States of America; 4 Walter Reed Army Institute of Research (WRAIR), Silver Spring, Maryland, United States of America; New York State Department of Health, UNITED STATES

## Abstract

Infectious diarrhea is a World Health Organization public health priority area due to the lack of effective vaccines and an accelerating global antimicrobial resistance crisis. New strategies are urgently needed such as immunoprophylactic for prevention of diarrheal diseases. Hyperimmune bovine colostrum (HBC) is an established and effective prophylactic for infectious diarrhea. The commercial HBC product, Travelan^®^ (Immuron Ltd, Australia) targets multiple strains of enterotoxigenic *Escherichia coli* (ETEC) is highly effective in preventing diarrhea in human clinical studies. Although Travelan^®^ targets ETEC, preliminary studies suggested cross-reactivity with other Gram-negative enteric pathogens including *Shigella* and *Salmonella* species. For this study we selected an invasive diarrheal/dysentery-causing enteric pathogen, *Shigella*, to evaluate the effectiveness of Travelan^®^, both *in vitro* and *in vivo*. Here we demonstrate broad cross-reactivity of Travelan^®^ with all four *Shigella spp*. (*S*. *flexneri*, *S*. *sonnei*, *S*. *dysenteriae* and *S*. *boydii)* and important virulence factor *Shigella* antigens. Naïve juvenile rhesus macaques (NJRM) were randomized, 8 dosed with Travelan^®^ and 4 with a placebo intragastrically twice daily over 6 days. All NJRM were challenged with *S*. *flexneri* 2a strain 2457T on the 4^th^ day of treatment and monitored for diarrheal symptoms. All placebo-treated NJRM displayed acute dysentery symptoms within 24–36 hours of challenge. Two Travelan^®^-treated NJRM displayed dysentery symptoms and six animals remained healthy and symptom-free post challenge; resulting in 75% efficacy of prevention of shigellosis (p = 0.014). These results strongly indicate that Travelan^®^ is functionally cross-reactive and an effective prophylactic for shigellosis. This has positive implications for the prophylactic use of Travelan^®^ for protection against both ETEC and *Shigella spp*. diarrheal infections. Future refinement and expansion of pathogens recognized by HBC including Travelan^®^ could revolutionize current management of gastrointestinal infections and outbreaks in travelers’ including military, peacekeepers, humanitarian workers and in populations living in endemic regions of the world.

## Introduction

Over 1.7 billion global cases of diarrheal disease and estimated 2.2 million deaths are reported annually, with the highest burden in low to middle income countries [1]. *Shigella* and enterotoxigenic *Escherichia coli* (ETEC) are amongst the most prevalent bacterial pathogens associated with diarrheal disease and a significant cause of mortality and morbidity worldwide [2–8]. Shigellosis is also an underreported problem in industrialized regions where outbreaks are not uncommon [9–11].

The increasing prevalence of emerging antimicrobial-resistant infections remains one of the greatest threats to global health, over the last several years, a dramatic rise in antibiotic resistance of enteric pathogens, including ETEC, *Shigella*, *Salmonella* and *Campylobacter spp*., has been documented in Southeast and South Asia [12–15]. With the onset of global antimicrobial resistance (AMR) alternatives to antibiotics are desperately needed [16]. Efforts to develop vaccines for prevention of acute infectious diarrhea has been a focus for more than 40 years with only partial success [17]. While an active vaccine is preferred, non-vaccine prophylaxis strategies may provide a reasonable level of protection and be an intermediate solution to the problem. One promising approach is oral administration of hyperimmune bovine colostrum (HBC) as an anti-diarrheal prophylaxis [18]. In some cases, HBC may provide an effective treatment alternative to antibiotics for gastrointestinal (GI) infections as *Clostridium difficile* in humans [19–21].

Colostrum is the first milk produced by mammals after birth and is rich in numerous immunoglobulins (Ig). Repeat immunization of pregnant dairy cows can stimulate production of high levels of vaccine-specific Ig in colostrum. When harvested, this HBC is enriched predominantly with vaccine antigen-specific IgG’s. Colostrum also contains lower levels of IgA and IgM, cytokines, growth factors, and antimicrobial peptides are also present [22–24]. In cows Ig’s are not transferred to the unborn calf during gestation, transmission only occurs *post-partum* via mammary secretions [25]. The content of bovine colostrum is enriched in immunoglobulin with levels of ≥ 50mg/ml, with IgG1 accounting for 85–95% of the total Ig concentration [25, 26]. Colostrum Ig’s are known to reside longer in the GI tract due to the presence of casein proteins and unlike antibiotics, do not adversely disrupt the resident microbiota [25, 26]. Passive oral administration of HBC has been evaluated as a preventive or therapeutic modality for a variety of enteric infections [27, 28], with the earliest published accounts involving treatment of cryptosporidiosis in human patients [29–32]. In addition, HBC was investigated as a passive immunotherapeutic agent against a wide variety of enteric microorganisms, including rotavirus [33–35], *Shigella* [36], ETEC [18, 37–41], *C*. *difficile* [19, 20, 26, 42, 43] and cholera toxin [44, 45].

Travelan^®^ is an ETEC-specific HBC (manufactured by Immuron Ltd., Australia) and commercially available for prophylaxis of travelers’ diarrhea (TD) over the counter in the United States, Australia, and Canada. Travelan^®^ is generated by immunizing cows with 13 different ETEC strains known to cause Travelers’ diarrhea: H10407 (O78:H11), B2C (O6:H16), C55 3/3c3 (O8:H9), PE 595 (O15:H4), E11881A (O25:H42), C1064-77 (O27:HR), PE 673 (O63:H-), E20738/0 (O114:H21), PE 724 (O115:H-), EI 37–2 (O128:H21), B7A (O148:H28), E8772/0 (O153:H12), PE 768 (O159:H-) [38].

The functional activity of Travelan^®^ was comprehensively reported in a publication by Sears and group [38] demonstrating, that Travelan^®^ contains high levels of antibodies against the target vaccine antigens O6 and O78 polysaccharides, CFA/I, CFA/II, CS3, CS4, CS6, and LT (measured by ELISA assay). In addition, Travelan^®^ also cross-reacts with ETEC O serotypes not included in the vaccines, including O44, O55, and O127, as well as *Shigella* and *Salmonella* O polysaccharides (measured by ELISA assay).

Travelan^®^ also contains cytokines, growth factors, and lactoferrin that can potentially provide innate immune defenses and promote intestinal tissue growth and repair [38]. Travelan^®^ and other similar HBC products have the capacity to bind and inhibit enteric pathogen motility in the GI tract, preventing attachment to the mucosal intestinal wall thereby neutralizing the ability to cause diarrhea and its associated symptoms [38, 46, 47]. Travelan^®^ represents a novel natural, and efficacious product to prevent TD with an excellent safety profile, demonstrated biological activity and clinical efficacy. Clinically, Travelan^®^ when taken orally thrice daily was shown to reduce both the incidence and severity of ETEC-induced diarrhea in controlled human infection models demonstrating up to 90.9% protection [18].

Other similar HBC or milk concentrated IgG have also been used to passively protect healthy adults against ETEC [37, 39, 48, 49].

Shigellosis (typically causes watery diarrhea followed by dysentery), caused by *Shigella* species, remains a common cause of diarrhea in young children from developing countries [50], in certain communal care settings [51, 52] and in travelers and military personnel [37, 53]. Infection with *Shigella* leads to a strong inflammatory response characterized by bacterial invasion of the epithelium, massive recruitment of neutrophils and extensive destruction of the epithelial lining during the acute phase [54]. Furthermore, infection with *Shigella* leads to a robust humoral immune response directed to lipopolysaccharide (LPS) and the invasion plasmid antigen (Ipa) proteins, IpaB, IpaC and IpaD, which renders protection against subsequent infection by the same *Shigella* serotype [55]. US Army Medical Directorate of the Armed Forces Research Institute of Medical Sciences (USAMD-AFRIMS) has an established non-human primate (NHP) *Shigella* challenge model [56]. The NHP *Shigella* model recapitulates the symptoms of shigellosis such as diarrhea, bloody, mucoidal stools and ulceration seen in rectal biopsies [54, 56, 57].

Humans and NHPs share susceptibility to many enteric pathogens, including *Shigella*, ETEC and *Campylobacter spp*., serving as invaluable tools for studying human infectious diseases [49, 58]. NHP are used to understand pathogenesis, induction of protective immunity and efficacy of products, such as vaccines and other countermeasures. Naïve juvenile rhesus macaques (NJRM) appear to be the most susceptible to this type of infection [59] and are a suitable substitute to model human clinical disease for evaluating anti-diarrheal interventions.

It has been shown that Travelan^®^ can recognize Gram-negative enteric pathogens besides ETEC [38]. To expand the observation that Travelan^®^ cross-reacts to *Shigella spp*., in this study a series of *in vitro* and *in vivo* studies were conducted. The cross-reactivity of Travelan^®^ with *Shigella spp*. was evaluated by Western blot analysis. The functional in vivo activity of Travelan^®^ and the immunoprophylactic ability to protect against *Shigella*-induced diarrhea was studied in an established NJRM model.

## Materials and methods

### Bacterial isolates

ETEC and *Shigella* strains were used to evaluate the *in vitro* immune-reactivity of Travelan^**®**^ (Lot 1510002493) and Promilk 85 (protein rich milk powder (85% pure milk protein), Tatura Milk Industries Ltd., Australia). Bacterial isolates (72 ETEC and 65 *Shigella* isolates) were collected from Africa, Argentina, Bangladesh, Bhutan, Brazil, Cambodia, Egypt, France, Nepal, Thailand and Turkey.

Analysis was also performed using well-characterized, historical isolates: ETEC H10407 (LT/ST/CFA/I), *S*. *flexneri* 2a 2457T, *S*. *flexneri* 3a J17B, *S*. *flexneri* 6CCH60 and *S*. *sonnei* Moseley. ETEC strains were grown on colonization factor antigen (CFA) agar plates with or without bile salts (as appropriate) [60].

Immunoblots were performed on all isolates and representative blots for ETEC strains expressing LT and ST toxins, CS1, CS2, CS3, CS4, CS5, CS6, CS7, CS14, CS17, CS19, CS21, PCFO71, CFA/I, and CF -negative isolates are shown.

*Shigella* strains included four species of *Shigella* (*S*. *flexneri*, *S*. *dysenteriae*, *S*. *boydii*, and *S*. *sonnei*). *Shigella* strains were initially grown on tryptic soy agar (TSA) plates and after that isolated colonies in Luria Bertani (LB) broth. In addition to *Shigella* strains, purified *Shigella* antigens were also used to evaluate the *in vitro* immune-reactivity of Travelan^**®**^ and Promilk 85.

### *Shigella* antigens

*Shigella* proteins IpaB, IpaC, IpaD, and LPS from *S*. *flexneri* 2a, *S*. *flexneri* 3a, *S*. *flexneri* 6, and *S*. *sonnei* Moseley were produced in house. Briefly recombinant clones harboring the Ipa proteins of interest were cloned in the pET15b system and expressed in BL21 *E*. *coli* as reported previously [61, 62]. The IpaB and IpaC proteins were purified via a histidine tagged IpgC chaperone protein. The chaperone protein was subsequently purified from the IpaB/IpaC. IpaD was purified as a GST tagged protein from recombinant *E*. *coli* and the GST tag was subsequently removed. Invaplex is a large, macromolecular complex consisting of the major *Shigella* antigens, Lipopolysaccharide (LPS) and the invasion plasmid antigen (Ipa) proteins B, C and D [55, 56, 63].

### Sodium dodecyl-sulfate polyacrylamide gel electrophoresis (SDS-PAGE) and immunoblot analysis

SDS-PAGE stained with Gel Code Blue or immunoblot analysis was performed with ETEC whole cell lysates [64], and with whole cell lysates of *Shigella* isolates and purified *Shigella* antigens [61, 63, 65]. Travelan^®^ immunoblot analysis of the whole cell lysates of ETEC strains were performed to confirm immunoreactivity of Travelan^®^ as previously it was done by ELISA. The freshly harvested bacterial cell pellet in Laemmli sample buffer was heated and centrifuged at high speed to remove cell debris, supernatants were loaded onto Mini-PROTEAN^®^ TGX™ Precast Protein Gels. Bio-Rad. Precision Plus Protein™ Dual Color Standards were used as molecular weight markers. Three gels were run simultaneously e, one gel was stained with Gel Code Blue, and the other two were immunoblotted. One immunoblot was probed with Travelan^®^ and the other was probed with Promilk 85 and bands were detected with alkaline phosphatase goat anti-bovine antibodies (SeraCare life sciences, Massachusetts, USA) as the secondary antibodies. Blots were developed using BCIP/NBT color development substrate (Promega, Wisconsin, USA) according to the manufacturer’s instructions, or SIGMA FAST^TM^ Fast Red TR/Naphthol AS-MX Alkaline Phosphatase Substrate (Sigma-Aldrich, Missouri, USA).

### Cross reactivity of Travelan^®^ to *Shigella* antigens

Cross reactivity of Travelan^®^ to *Shigella* antigens was also measured by ELISA. Travelan^®^ and Promilk 85 were rehydrated in PBS-0.05% Tween 20 at 16 mg/mL total product weight as previously described [38]. Four *Shigella* LPS antigens (*S*. *flexneri* 2a, *S*. *flexneri* 3a, *S*. *flexneri* 6 and *S*. *sonnei*) and three Ipa proteins (IpaB, IpaC and IpaD) antigens were coated at 1 μg/mL onto Immulon 1B 96 well plates (Thermo Fisher Scientific) [63]. Mouse monoclonal antibodies specific for each antigen were used as positive controls, except a rabbit polyclonal serum was used for detection of *S*. *flex* 6, and Promilk 85 was used as the negative control. Coated wells were blocked with 1% Blocker Casein buffer (Thermo Fisher Scientific). Travelan^®^, Promilk 85 and positive controls were analyzed in duplicate and diluted in 0.5% casein-PBS buffer. Specific antibodies were detected using HRP conjugated anti- bovine, mouse, or rabbit IgG, followed by 3,3’, 5,5’-tetramethylbenzidine (TMB) microwell peroxidase substrate. Absorbance values at 450 nm were measured on a microplate spectrophotometer (Thermo Fisher Multiskan FC) and analyzed by 4PL regression (GraphPad Prism). Endpoint titers were calculated as the inverse of the sample dilutions that produced an absorbance value at 450 nm (*A*450) of 3 times the average blank.

### Efficacy assessment of Travelan^®^ for prevention of shigellosis in the NJRM model

Ethical approval to conduct the study was provided by the USAMD-AFRIMS Institutional Animal Care and Use Committee. The study was conducted by the Department of Veterinary Medicine (DVM), accredited by the Association for the Assessment and Accreditation of Laboratory Animal Care, International (AAALAC), in accordance with the “Guide for the Care and Use of Laboratory Animals” published by the National Research Council, 2011, 8th Edition.

Naïve juvenile rhesus macaque (*Macaca mulatta)* selected for the study were negative for tuberculosis (TB), simian immunodeficiency virus (SIV), simian T-cell lymphotropic virus type 1 (STLV-I), and type-D retrovirus (SRV-D). All NJRM were in good health, as shown by physical examination (PE), complete blood counts (CBC), and blood chemistry profiles. Animal activity, feed consumption, and other clinical observations were recorded twice daily during the study.

NJRM are born and raised in paired then group housing within the facility which has insect screens enabling exposure to the environmental conditions in Thailand. An enrichment program is focused on providing a stimulating environment using the latest industry accepted practices. Veterinary staff are available on site 24/7, and all animals are observed at least twice per day. Animals are fed a commercial primate feed that is supplemented with locally procured produce and commercially available vitamins as directed by veterinary staff. While on study, animals are confined in single or double cages in a separate wing of the facility that receives HVAC control for temperature and humidity that is continuously monitored by an automated system.

Twelve NJRM were screened for inclusion in this study using the following criteria: age within 3–5 years old, weight ≥ 3.2 kg; serum anti-*S*. *flexneri* 2a LPS specific IgA antibody titers ≤ 200; IgG and IgM antibody titers ≤ 400; no diarrheal symptoms for 14 days prior to study initiation; good health; CBC and blood chemistry values within DVM assigned normal limits. After selection, NJRM were treated with enrofloxacin antibiotic for 5 consecutive days as a prophylactic measure to minimize any pre-existing diarrheal diseases, which may be caused by the heavily populated gut lumen with various bacterial strains.

### Prophylaxis study

Two weeks after antibiotic treatment, 12 NJRM were randomly assigned into two groups, 8 received Travelan^®^ (Lot # 1510002493) treatment and 4 received the Promilk 85 as a placebo control. NJRMs were fasted overnight and anesthetized with ketamine hydrochloride before nasogastric placement. Travelan^®^ was delivered intragastrically twice daily, 12 hrs apart, for a total of 12 doses over 6 days (study days (SD) 0 to 5). Similarly, control NJRM received Promilk 85 as a placebo for 6 days using the same schedule. On SD 3 all NJRM had received challenge dose (2.76x10^9^ CFU) of *S*. *flexneri* 2a 2457 T strain [56].

Travelan^®^ and Promilk 85 solution was prepared daily as a 500 mg/dose, using water for injection (WFI). Each inoculation was administered after a CeraVacx buffer (Cera Products Inc., South Carolina, USA) administration [66, 67].

*S*. *flexneri* 2a, 2457T was grown in Luria-Bertani broth (BD Difco^TM^) and the bacterial suspension in PBS (Diamedix, OH, USA) was adjusted to the appropriate OD_600_ corresponding to 2–5 x10^8^ CFU/ml. Approximately 10 ml of the challenge inoculum was administered on study day 3 to all NJRM.

### Disease assessment

NJRM were monitored during the study for any symptoms that suggested an adverse reaction, including diarrhea, fever, vomiting, reduced activity and recumbency, allergic reactions (e.g., swelling, itchiness, and rash), or anaphylactic reactions (e.g., collapse, anemia, hypotension, tachypnea, dyspnea, hypothermia, tremors, seizure, and urinary incontinence). Animals were observed frequently and offered medical intervention when indicated by their clinical condition. All interventions requiring animal handling were performed under anesthesia.

All NJRM were closely observed and scored at least twice daily according to the previously defined criteria [56, 68]. The observed clinical parameters were activity (active/ reduced/ immobile/ recumbent), appetite (≥90%/ ≥70%/ ≥50%/ ≤50%), fecal consistency (normal (score 1)/ soft (score 2)/ loose (score 3)/ watery (score 4)), fecal- mucus and/or blood (absent/ only mucus/ only blood/ both mucus and blood), and skin turgor (normal/ return 2–4 sec/ return 5–9 sec/ >10 sec return), as described earlier [56]. If the total clinical score was ≤ 4 = the NJRM was in normal health; 5–9 = needed to be monitored carefully and might provide electrolytes in the drinking water; 10–14 = electrolytes in the drinking water or sub-cutaneous normal saline/ intra-venous infusion of acetated Ringer’s with or without 5% dextrose solution and analgesics considered (Metoclopramide [N-diethylamino ethyl)-2-methoxy-4-amino-5-chlorobenzamide] to relieve the symptoms of vomiting and Brupenex to treat pain); and ≥ 15 = NJRM euthanized. Under some circumstances, NJRM were euthanized with lower clinical scores as well. Dysentery for this study was defined as at least one watery or loose stool containing blood and mucus. All NJRM were humanely euthanized by intravenous pentobarbital injection at the end of all experimental procedures on day 13/14 or earlier if they met humane endpoint criteria.

### Shedding of *Shigella* challenge strain

Shedding of the challenge strain (*S*. *flexneri* 2a 2457T) was evaluated by standard bacterial culture method. After euthanasia, fecal materials were collected from different portions of the GI tract corresponding to the stomach, duodenum, cecum, jejunum, ileum, colon (ascending, transverse and descending), rectum and evaluated by culture methods.

### Assessment of inflammatory responses: Cytokine and biomarker levels and histological examination

The inflammatory response induced by *S*. *flexneri* 2a 2457T strain in all challenged NJRM were assessed using multiple measures. Pro-inflammatory cytokines (IL-1β, IL-6, and IL-8 (chemokine CXCL8)) and fecal biomarkers (calprotectin and myleoperoxidase) were measured in fecal extract samples using Milliplex^Ⓡ^ Map Non-Human Primate Cytokine Magnetic Bead Panel Kits (EMD Millipore Corporation, MA, USA) and analyzed by a MAGPIX Multiplex Reader or by using a commercial ELISA kit (Epitope Diagnostics Inc., California, USA).

Tissue samples from different portions of the GI tract including jejunum, ileum, cecum, colon (ascending, transverse and descending) and rectum were collected from euthanized/deceased animals in 10% neutral buffered formalin, processed routinely, and stained with standard hematoxylin and eosin stain (H&E) for a descriptive morphologic diagnosis. All slides were coded and interpreted and scored blindly by a board-certified veterinary pathologist, as previously reported [56, 69]. The severity of inflammation in the tissue sections was scored on a scale of 0–4: 0 (none); 1 (minimal); 2 (mild); 3 (moderate); 4 (severe). Severity scoring was a subjective assessment combining the extent of the necrotic lesion (how much of the tissue was affected) with how much damage, inflammation, or tissue occurred.

### Immune responses to *S*. *flexneri* 2a 2457T challenge strain

Promilk 85 treated NJRM serum samples were collected on SD: 3, 6, 8 and analyzed for antibody titers. Travelan^**®**^ treated NJRM serum samples were collected on SD: 6 and 8 (from early euthanized NJRM), and on day 10 for IgA and IgM and day 14 for IgG antibody titer measurements for the remaining NJRM.

Serum IgA, IgG and IgM antibody titers against *S*. *flexneri* 2a 2457T strain LPS and *S*. *flexneri* Invaplex were evaluated by ELISA as previously described [69]. I Antibody titers were presented as the fold increase of peak antibody titers over the baseline level, and seroconversion was defined as a ≥ 4-fold increase over baseline titers measured on day 0 the starting day of Travelan^**®**^/Promilk 85 treatment.

### Data analysis

Data analyses were performed using IBM SPSS Statistics, version 26 and Graph Pad Prism. The cytokine concentrations of the samples were calculated using ELISA Plus software, version 3.01 (MedData Inc., New York, USA). Data was statistically analyzed using where appropriate either nonparametric Chi-squared or Mann Whitney U analysis. Data showing statistical significance was noted in the text and Figures where statistical significance is defined as p < 0.05.

## Results

### *In vitro* reactivity of Travelan^®^ with ETEC strains

**Fig 1:**
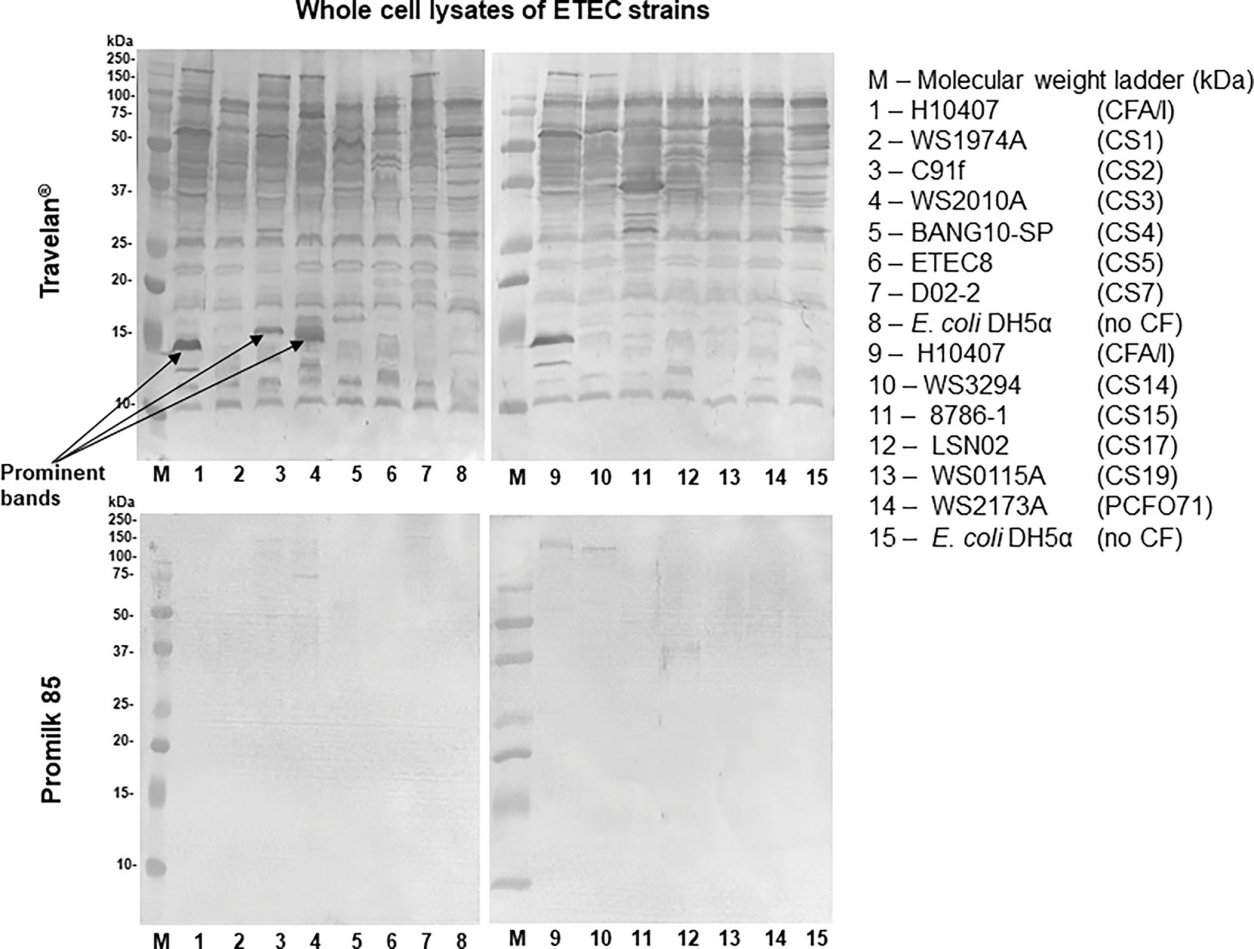
Travelan^®^ is reactive with a wide range of ETEC strains. Whole cell lysates prepared from a variety of ETEC strains representative of the major antigens CS1, CS2, CS3, CS4, CS5, CS6, CS7, CS14, CS17, CS19, CS21, PCFO71, CFA/I, and CF -negative isolates were analyzed by SDS-PAGE and western blotting followed by immunodetection with Travelan^®^ or ProMilk 85. Arrows show prominent protein bands.

Travelan^**®**^ is produced by immunizing cows with 13 ETEC strains, we first confirmed *in vitro* reactivity of Travelan^**®**^ to ETEC whole cells, from a diverse collection of ETEC strains. We have shown representative blots with strains expressing the major antigens LT and ST toxins, CS1, CS2, CS3, CS4, CS5, CS6, CS7, CS14, CS17, CS19, CS21, PCFO71, CFA/I, that included some of the strains/serotypes used for immunization. The SDS-PAGE patterns of all ETEC strains were similar (representative data is shown in Fig 1). Western blot analysis of the whole cell ETEC lysates with Travelan^**®**^ showed that antibodies in Travelan^**®**^ reacted with many proteins consistent with the use of whole ETEC cells as the vaccine antigen, as well as *E*. *coli* DH5α strain, which do not express CF antigens. ETEC strains expressing high levels of CFA/l (lane 1), CS2 (lane 3) and CS3 (lane 4) when probed with Travelan^**®**^ (Fig 1) showed prominent bands with approximate size range 13–17 kDa. Background binding was observed with the Promilk 85 blot, weakly detected only a few higher molecular weight proteins indicating insignificant or background binding.

### *In vitro* reactivity of Travelan^®^ with *Shigella* spp. cell lysates and *Shigella* virulence factor antigens

**Fig 2:**
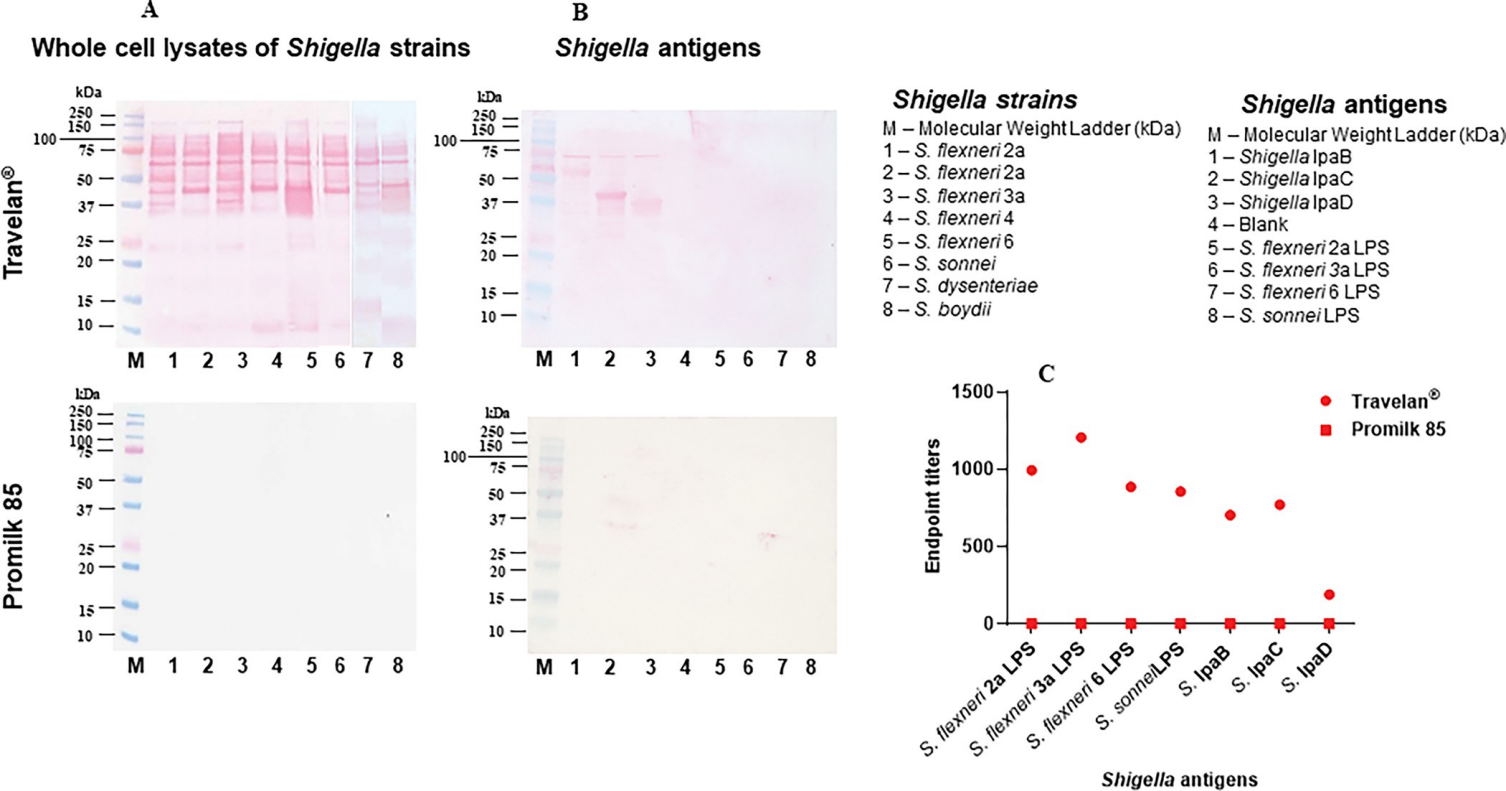
Travelan^®^ antibodies cross-react with all 4 *Shigella* species and major *Shigella* antigens. (A) Immunoblotting of whole cell lysates of *Shigella* strains and (B) *Shigella* antigens (IpaB, IpaC and IpaD proteins and *S*. *flexneri* 2a, *S*. *flexneri* 3a, *S*. *flexneri* 6 and *S*. *sonnei* LPSs) probed with Travelan^®^ and Promilk 85 was used as control. (C) Immunoreactivity of Travelan^®^ with *Shigella* LPS and Ipa antigens compared to Promilk 85 analyzed by ELISA. Results are expressed as endpoint titer against the antigens for Travelan^®^ and Promilk 85.

Whole cell lysates of *Shigella* species were analyzed by Western blot with Travelan^®^ and Promilk 85. Antibodies in Travelan^**®**^ reacted with many protein bands in the whole cell lysate preparations and among these there were several prominent bands of apparent molecular weight ~65kDa, ~45kDa and ~40 kDa (representative data is shown in Fig 2A). Similarly, Travelan^®^ antibodies showed reactivity to recombinant Ipa proteins IpaB, IpaC and IpaD, with the highest binding to IpaC (Fig 2B). However, by Western blot analysis, all 4 *Shigella spp*. LPSs were not detected by Travelan^**®**^. The blot was repeated and probed with specific antibodies to ensure all LPS proteins were effectively transferred onto the nitrocellulose membrane, however after 3 repeats it was confirmed that no binding of this preparation of Travelan^®^ was observed.

### Immunoreactivity of Travelan^®^ against *Shigella* Ipa proteins and LPSs antigens by ELISA

To further characterize the immunoreactivity of Travelan^®^, antibody titers specific to *Shigella* LPSs (*S*. *flexneri* 2a, *S*. *flexneri* 3a, *S*. *flexneri* 6 and *S*. *sonnei* Moseley) and Ipa proteins (IpaB, IpaC and IpaD) were measured by ELISA. The endpoint IgG titers of Travelan^®^ and Promilk 85 are shown in Fig 2C. Travelan^®^ antibodies reacted strongly with all four species of LPS and recombinant proteins and titers were slightly lower to Ipa proteins IpaB and IpaC and approximately 4-fold lower reactivity was observed for Travelan^®^ antibody binding to IpaD. No *Shigella*-specific IgG responses were detected in Promilk 85 by ELISA. These results clearly indicate that antibodies present in Travelan^®^ are cross-reactive against major virulent *Shigella* antigens.

It is unclear why Travelan^®^ antibodies were reactive with LPS antigens in ELISA but not by immunoblotting. This may relate to protein conformation in the presence of SDS hindering accessibility of epitopes or because ELISA is generally more sensitive than western blotting.

### Travelan^®^ protects NJRM against shigellosis following oral challenge of with *S*. *flexneri* 2a 2457T

Prior to administration of the challenge strain clinical monitoring revealed no immediate adverse reactions including diarrhea, loose, or watery stool, vomiting, inactivity or posturing associated with pain or illness was observed in any of the NJRM during study days (SD) 0 to 2. This indicates Travelan^®^ or Promilk 85 treatment was safe and well tolerated in NJRM.

Each NJRM received clinical scores daily (based on presence of clinical symptoms) summarized in Table 1. Occurrence of clinical symptoms, such as vomiting, diarrhea, dysentery, activity level, medication and dehydration treatment was noted between the two groups on each SD.

**Table 1:** NJRM clinical symptoms and clinical score after challenge with live *S*. *flexneri* 2a 2457 T strain. Daily monitoring of symptoms from Study Day (SD) 4 (1 day post challenge) for all NJRM (M1-M12) to SD 14 (11 days post challenge).

NJRM # Group	Study Day (SD) Symptoms and Clinical Score (CS)													
	SD 4		SD 5		SD 6		SD 7		SD 8		SD 9–12		SD 13–14	
	Symptoms & Med	CS	Symptoms & Med	CS	Symptoms & Med	CS	Symptoms & Med	CS	Symptoms & Med	CS	Symptoms & Med	CS	Symptoms & Med	CS
M1 Placebo	D, V, SC, M	**8**	V, D	**6**	Dys, E	**10**	Dys, RA, SC, B, M	**8**	Dys, RA	**12** **Euthn**				
M2 Placebo	D, SC	**6**	Dys, De, SC,	**10**	Dys, RA, De	**12** **Euthn**								
M3 Placebo	D, RA, IV, De	**7**	Dys, V, RC, NR, B, De, IV,	**15** **Died**										
M4 Placebo	D, V, M, SC	**8**	Dys, V, M, SC,	**12**	Dys, NR, De	**15** **Euthn**								
M5 Travelan^®^	D, E	**6**	Healthy	**5**	Healthy	**3**	Healthy	**3**	Healthy	**3**	Healthy	**1**	Healthy	**<1**
M6 Travelan^®^	Healthy	**5**	Healthy	**2**	Healthy	**1**	Healthy	**1**	Healthy	**2**	Healthy	**<1**	Healthy	**<1**
M7 Travelan^®^	Healthy	**3**	Healthy	**3**	Healthy	**3**	Healthy	**3**	Healthy	**5**	Healthy	**1**	Healthy	**<1**
M8 Travelan^®^	Healthy	**5**	Healthy	**3**	Healthy	**3**	Healthy	**3**	Healthy	**3**	Healthy	**2**	Healthy	**<1**
M9 Travelan^®^	Healthy	**3**	Healthy	**3**	Healthy	**3**	Healthy	**3**	Healthy	**3**	Healthy	**1**	Healthy	**<1**
M10 Travelan^®^	V, E, M	**6**	Healthy	**5**	Healthy	**3**	Healthy	**3**	Healthy	**3**	Healthy	**<1**	Healthy	**<1**
M11 Travelan^®^	D, E, M	**6**	D, IV, B	**8**	Dys, RC	**14** **Euthn**								
M12 Travelan^®^	V, E, M	**6**	V, Dys, SC	**8**	Dys, RA, SC, B, M	**9**	Dys, V, RA, IV, B	**11**	Dys, De, RA	**11** **Euthn**				

Symptom abbreviations: D = Diarrhea, V = Vomiting, Dys = Dysentery, De = Dehydration. Activity level: RA = Reduced activity, RC = Recumbent, RC = Non-responsive and inactive. Medication (Med): M = Metoclopramide treatment for nausea, B = Buprenex to treat pain. Dehydration treatment: E = electrolytes added to water bottle, SC = subcutaneous saline solution, IV = intravenous infusion of acetated Ringer’s with or without 5% dextrose solution. Clinical score: ≤4 = Normal health, 5–9 = monitored carefully electrolytes added to water, 10–14 = SC or IV and/or analgesics and euthanasia considered, ≥15 = Euthn = Euthanized.

Clinical parameters (healthy, vomiting, diarrhea, dysentery, activity level, dehydration) were compared between two groups using Chi-squared analysis (statistically significant at p<0.05). Diarrhea on SD4 was significantly higher in the Promilk 85 group compared to the Travelan^®^ group (p = 0.01). On SD5, vomiting and dysentery were significantly higher (p = 0.03, for both symptoms) in the Promilk 85 group. The average clinical score was significantly higher (p = 0.02) in the Promilk 85 placebo group compared to the Travelan^®^ group and the average fecal consistency score was significantly higher (p = 0.002) in the Promilk 85 group compared to the Travelan^®^ group.

On SD3 (4^th^ day of Travelan^®^ or Promilk 85 treatment), all NJRM were challenged with *S*. *flexneri* 2a, 2457T, there were no clinical symptoms on SD3.

On SD4 (day 1 post-infection), all 4/4 NJRM (M1-M4) in the Promilk 85 placebo group had watery stools with mucus, and 2/4 vomited. In the Travelan^®^ treated group 2/8 animals vomited (M10, and M12), and M5 and M11 had loose stools with mucus. All animals affected with vomiting received electrolytes in water bottles, SC or IV and medications as detailed in Table 1. Diarrhea on SD4 was significantly higher in the Promilk 85 group compared to Travelan^®^ group (p = 0.01, Chi-squared test).

On SD5 (last day of Travelan^®^ or Promilk 85 treatment), vomiting and dysentery were significantly higher (p = 0.03, Chi-squared test, for both symptoms) in the Promilk 85 group. 3/4 NJRMs in the placebo group had dysentery. Two of the NJRM with dysentery and the one with diarrhea vomited. One of this group M3 died whilst being assessed for euthanasia on SD5 with severe symptoms, low body temperature, abdominal cramping and no movement with a clinical score of 15. In contrast 1/8 NJRM in the Travelan^®^ group had diarrhea (M11) and one had dysentery (M12). The two NJRM treated with Travelan^®^ who suffered from diarrhea on SD4 (M5) and vomiting (M10) by SD5 had recovered and had clinical scores of 5.

On SD 6 (3 days after challenge), two NJRMs in the Promilk 85 group (clinical scores of 12 & 15) and one NJRM in the Travelan^®^ group (clinical score 14) were euthanized by the attending veterinarian. One NJRM in the Travelan^®^ group had dysentery and reduced activity the remaining 6/8 NJRM in the Travelan^®^ group remained healthy (clinical scores 1–3).

On SD 7 the remaining animal in the Promilk 85 group and one of the symptomatic animals in the Travelan^®^ group were severely dehydrated and suffering from dysentery and on SD 8 these two animals were euthanized. The remaining 6/8 animals in the Travelan^®^ group remained healthy throughout the remainder of the study to SD13-14.

**Fig 3:**
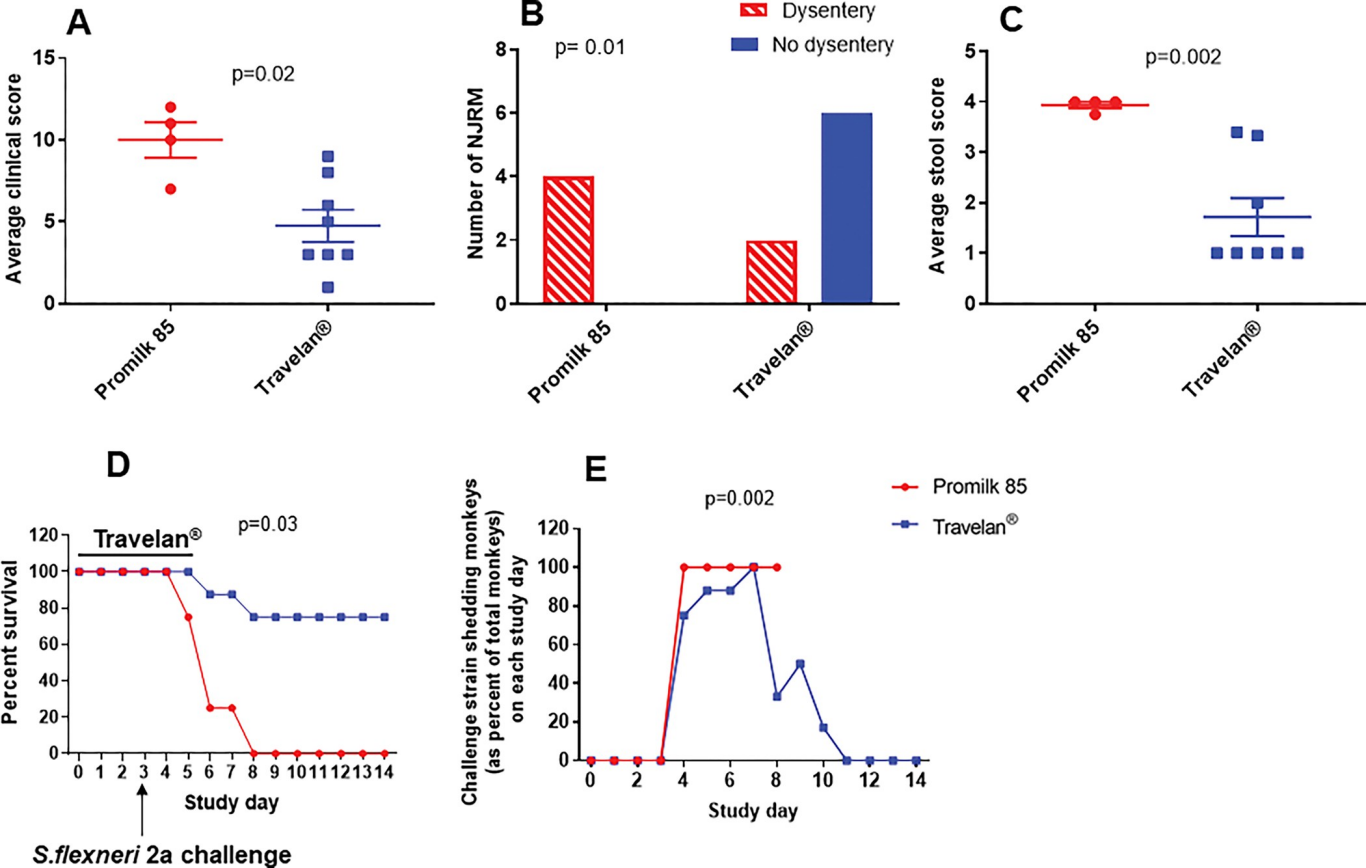
Clinical symptoms, survival and *S*. *flexneri* 2a shedding. (A). Average clinical score for each animal in the Travelan^®^ and Promilk 85 groups over the course of post-infection monitoring Daily scores were averaged for each animal until euthanasia/death and represented as individual values. Mean values are represented as a horizontal line and SEM displayed as error bars, significance between groups was analyzed by Mann Whitney U statistics. (B) Number of animals in the Travelan^®^ or placebo groups with dysentery/shigellosis following *S*. *flexneri* 2a challenge analyzed by Chi squared statistics. (C). Average fecal consistency (diarrhea) score in placebo and Travelan^®^ groups. Daily stool scores were averaged for each animal until euthanasia/death and represented as individual values. Mean of the group is shown by a horizontal line and SEM shown by error bars and significance between groups analyzed by Mann Whitney U statistics (D).Survival of NJRMs in the Travelan^®^ and the Promilk 85 placebo group, measured daily as a percentage. Statistics was measured by Chi squared analysis (E) Shedding of *S*. *flexneri* 2a challenge strain as percent of survived monkeys in the Promilk 85 and Travelan^®^ groups analyzed by Mann Whitney U statistical analysis.

To compare the clinical score post-infection for each group, the daily clinical score per animal over the post infection period until euthanized was averaged. The average clinical score was significantly higher (p = 0.02) in the Promilk 85 placebo group compared to Travelan^®^ group (Fig 3A). The Travelan^®^ group had fewer clinical symptoms which indicates Travelan^®^ offered protection against the onset of diarrheal symptoms post-challenge.

Over the course of the post-infection monitoring period, all four of NJRM in the Promilk 85 group developed dysentery (M1-M4), whereas only 2 of 8 animals in the Travelan^®^ group (M11-M12) developed dysentery with 6 of 8 being protected from dysentery (M5-10), as shown in Fig 3B.

The average fecal consistency score was significantly higher in the Promilk 85 group compared to the Travelan^®^ group, further demonstrating that Travelan^®^ protected animals from developing *Shigella*-mediated diarrhea (Fig 3C).

Travelan^®^ treatment prolonged the survival of NJRM compared to those receiving the placebo Promilk 85 treatment (Fig 3D). NJRM (6/8) receiving Travelan^®^ were protected from dysentery symptoms and remained healthy resulting in 75% protective efficacy and this was statistically significant (p = 0.014, Chi squared Test).

This study indicates that the active antibody components of Travelan^®^ are functionally cross-reactive against *S*. *flexneri* 2a and afford protection against shigellosis in the NJRM model.

### Travelan^®^ prophylaxis treatment reduces *Shigella* colonization

To assess the clearance of the challenge strain, the levels of *S*. *flexneri* 2a shedding were measured by stool cultures after challenge. All NJRM in the Promilk 85 control group were *Shigella*-positive until they were euthanized or died. Shedding was significantly lower in the Travelan^®^ treated group compared to the Promilk 85 treated group (Fig 3E) measured as the number of animals who shed challenge strain (as percent of total animals) on each study day.

To further assess effects of treatment on colonization, stool samples present in different sections of the large and small intestine were collected and cultured during dissections following early or planned termination in severely symptomatic or healthy animals, respectively. For all four Promilk 85 treated animals and the two symptomatic Travelan^®^ treated animals, fecal materials from different parts of GI tract were shown by culture to all be positive for *S*. *flexneri* 2a on termination. Conversely, the six healthy Travelan^®^ treated animals were all shown to have negative GI tract *S*. *flexneri* cultures at termination. This indicates the positive effects of Travelan^®^ prophylaxis in assisting with clearance of the challenge strain.

### Travelan^®^ protects against *Shigella* induced gut inflammation

The inflammatory changes induced by challenge with *S*. *flexneri* 2a, 2457T in all NJRM was assessed by measuring levels of inflammatory cytokines IL-1β, IL-6, IL-8, calprotectin (a fecal protein marker for inflammation) and Myeloperoxidase (MPO; an enzyme associated with inflammation) in fecal stool samples collected on SD 0, 1, 3, 5, 7, 9, 11 and 13.

The peak fecal cytokine levels in animals treated with Promilk 85 versus Travelan^**®**^ was compared, however due to the 2 out of 8 animals suffering from diarrheal symptoms in the Travelan^®^ treated group, the groups were modified into the following two groups: (i) ‘Protected’ all animals who did not encounter severe symptoms, and (ii) ‘Not-protected’ animals suffering from adverse symptoms, i.e., those in the placebo group plus two of the Travelan® group (M11 and M12) who displayed dysentery symptoms.

**Fig 4:**
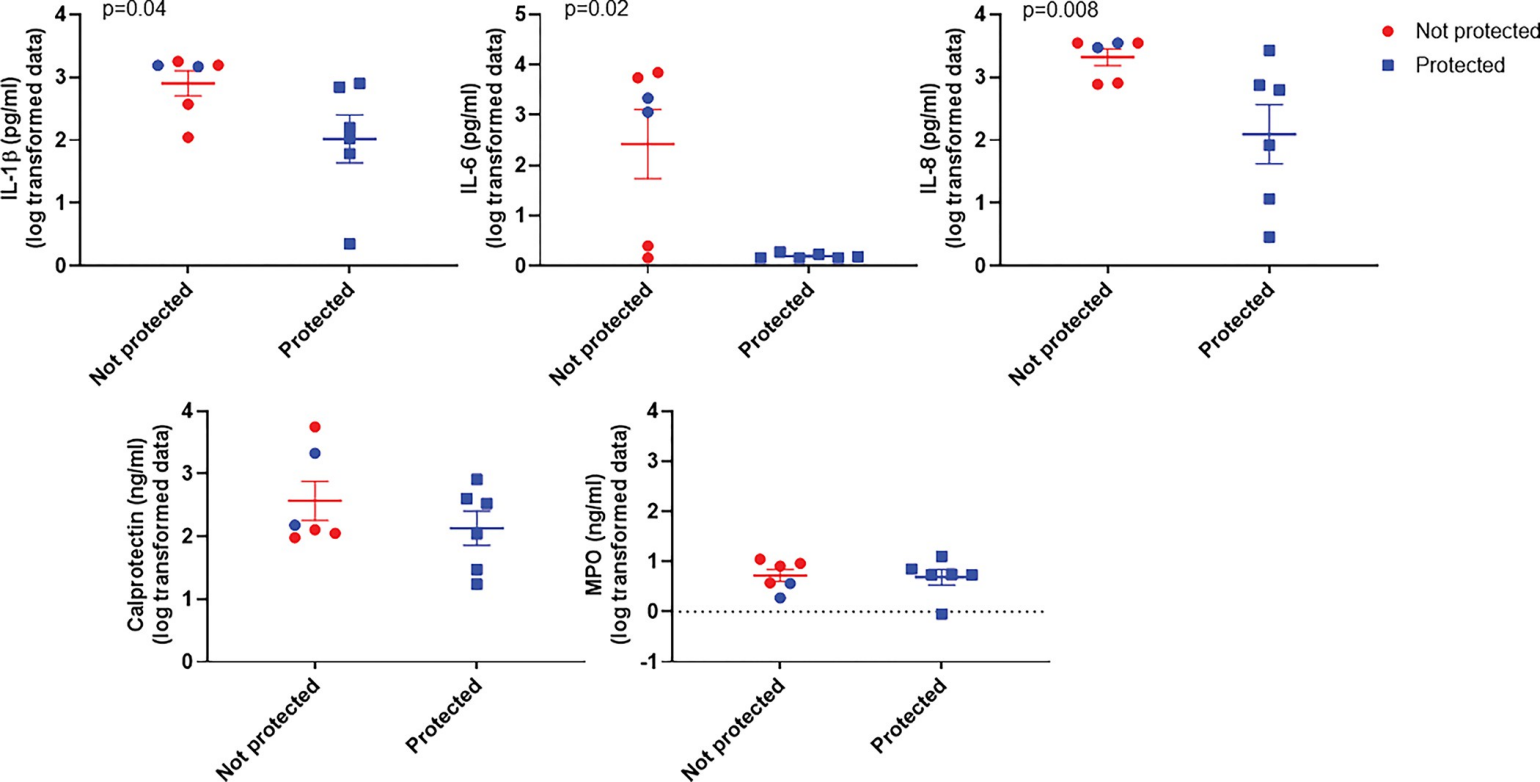
Analysis of inflammatory cytokines and biomarkers in fecal samples. NJRM were grouped into a ‘not protected’ group including the animals who suffered from dysentery symptoms i.e., all 4 animals in the Promilk 85 placebo group (red circles) plus two of the Travelan^®^ group were not protected (blue circles). The second group denoted ‘protected’ group included the 6 animals not suffering from symptoms all in the Travelan^®^ group (blue squares). The circle/square points represent the peak cytokine levels per animal, the mean level is shown by a horizontal line and SEM as the error bars. Statistical analysis (Mann Whitney U) levels of significance are shown.

Cytokine and inflammatory markers levels for the not-protected and protected groups is shown in Fig 4. Pro-inflammatory cytokine levels were significantly higher (p<0.05) in the ‘Not protected’ group for IL-1β, IL-6 and IL-8 compared to the ‘Protected’ animals. However, there was no significant difference (p>0.05) in the levels of fecal MPO and calprotectin biomarker expression in the ‘Protected’ group who received Travelan^®^ compared with the ‘non protected’ group.

### Intestinal damage measured by histology is reduced in Travelan^®^ treated NJRM

To further examine the effects of *Shigella* infection on the GI tract and to investigate if Travelan^®^ provided any beneficial protective effects, histological samples were collected from euthanized animals and analyzed. Gut tissue sections: jejunum, ileum, cecum, colon (ascending, transverse and descending) and rectum were analyzed for necrosis, hemorrhage, edema, ulceration or erosion of the epithelium, or lymphoid necrosis of the gut-associated lymphoid tissue. Inflammation following challenge was categorized by the presence of neutrophils, lymphocytes, plasma cells, lymphoplasmacytic, and macrophages. Proliferation was noted as either being present or absent, and included epithelial hyperplasia and regeneration.

The microscopic findings in the NJRM displaying dysentery symptoms included tissue damage in the rectum characterized by proctitis, necrotizing, diffuse (epithelial and submucosal), acute, marked with fibrin, hemorrhage, and edema; and in the colon characterized as colitis, necrotizing, proliferative and lymphoplasmacytic diffuse (epithelial and submucosal), acute, marked with fibrin, hemorrhage, and edema, multifocal epithelial necrosis, erosions, villas blunting, fusion, and loss; hemorrhage, and suppurative lymphoid necrosis.

**Fig 5:**
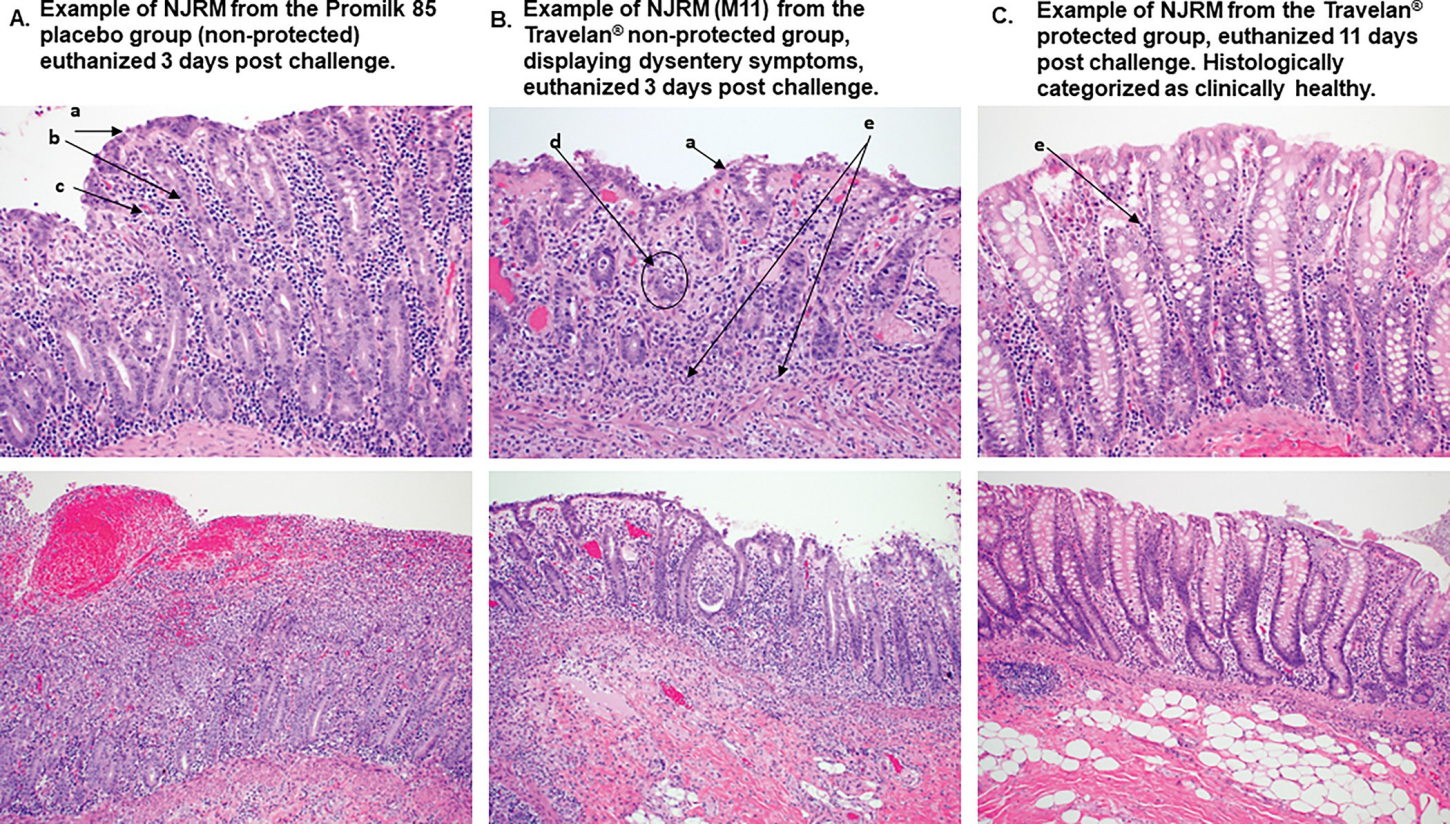
Histological evaluation of NJRM after challenge. Representative H&E staining images of colon sections from shigella- infected NJRM animals (A) NJRM from the Promilk 85 placebo group with highest colon and rectum severity score of 3–4, displaying all histological changes a-e (refer to list below). (B) NJRM #M11 from the Travelan^**®**^ group who displayed dysentery symptoms. Histologically had a moderate severity score in the colon and rectum of 2. Displaying histological changes: a, d and e. (C) NJRM from the Travelan^**®**^ group who did not display dysentery symptoms. Histologically tissue was shown to be healthy with a minor change (e). Histological changes observed: a) villi blunting, fusion and loss; b) crypt hyperplasia with degenerative neutrophils; c) fibrin, hemorrhage and edema; d) crypt abscess with necrotic debris and degenerative neutrophils; e) infiltration of neutrophils. Examples are shown by arrows. Bottom panels show inflammatory infiltrate at 100x and upper panels at 200x.

In the NJRM treated with Promilk 85 who died within 72 hrs of challenge, pathology findings were more severe the rectum had proctitis, necrotizing, multifocal, acute, moderate with mixed inflammation; colon (ascending, transverse, and descending): colitis, proliferative and lymphoplasmacytic, diffuse, subacute, mild to moderate, with multifocal epithelial necrosis, erosions, villar blunting, fusion, and loss. The ascending colon and rectum were the most affected areas in the Promilk 85 treated animals (Fig 5A).

There was no evidence of inflammation in the colon in any of the Travelan^®^ group who did not develop dysentery, and only one animal displayed minor infiltration of neutrophils in the ileum (Fig 5C). Two of eight Travelan^®^ treated animals became symptomatic, their histological analysis revealed less severe damage compared to all of the Promilk 85 group. An example is shown in Fig 5B, the NJRM receiving Travelan^®^ with dysentery symptoms (M11) had a histological severity score of 2, this image indicates much lower tissue inflammation than in the Promilk 85 treated animal (Fig 5A) displaying severe histological changes.

**Fig 6:**
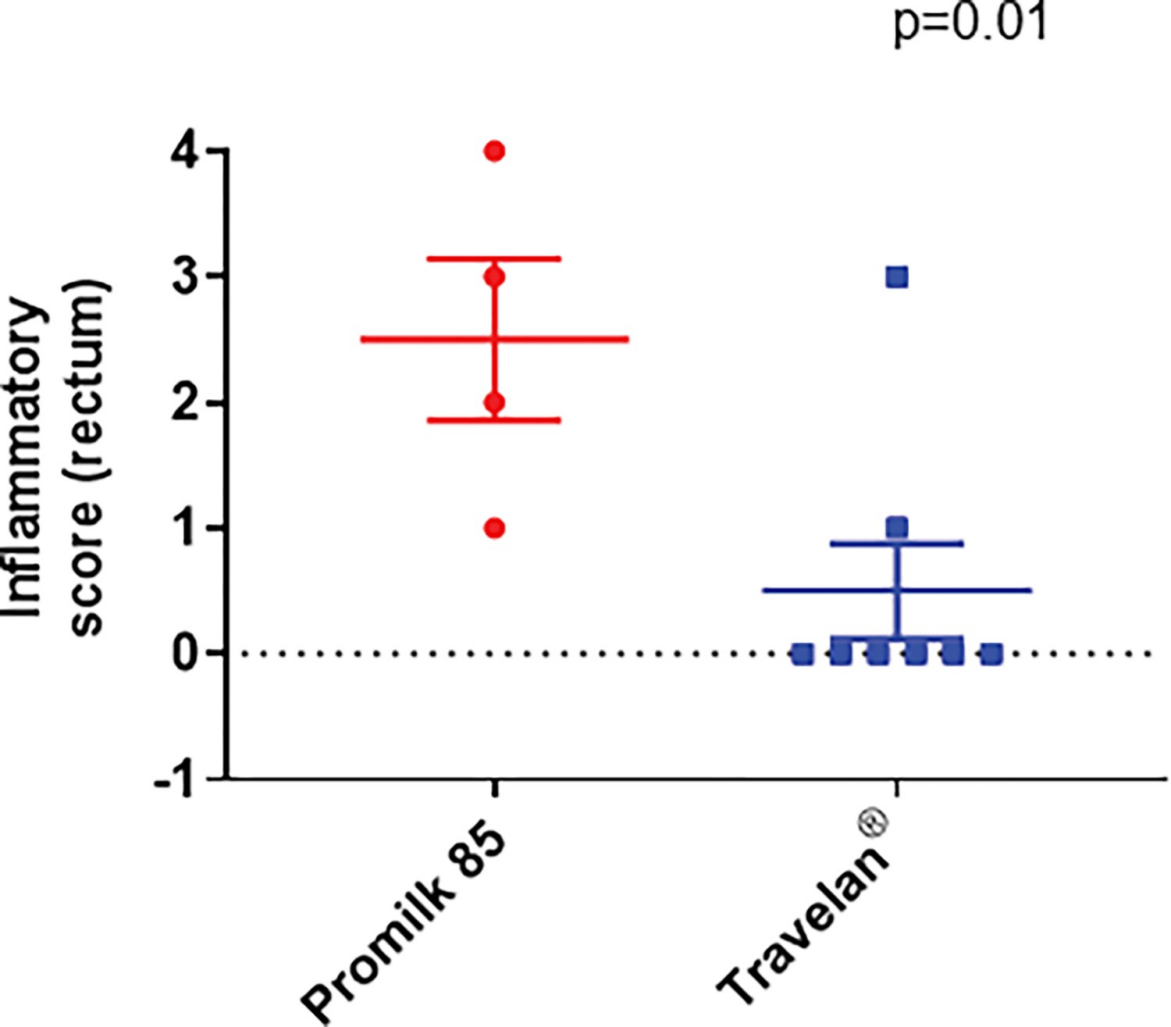
Histological severity (inflammatory) score in the rectum of the GI tract of NJRM post *Shigella* challenge. Histological samples for all NJRMs were analyzed and scored (severity score levels 0–4). The values are plotted per animal and the mean values are shown as a horizontal line and SEM as error bars. Mann Whitney U statistical analysis was performed.

The severity score between the Travelan^®^ and the Promilk 85 groups for rectal histology was measured and found to be significantly higher for the placebo group (Fig 6 (p = 0.01, Mann-Whitney U analysis).

These results indicate Travelan^®^ blocks *Shigella* colonization in the gut, thereby reducing inflammation, measured by the reduction in inflammatory cytokines in Fig 4 and the reduction in histological colon and rectal severity score in Figs 5 and 6.

### *Shigella* immunity is not hindered by Travelan^®^ prophylaxis

**Fig 7:**
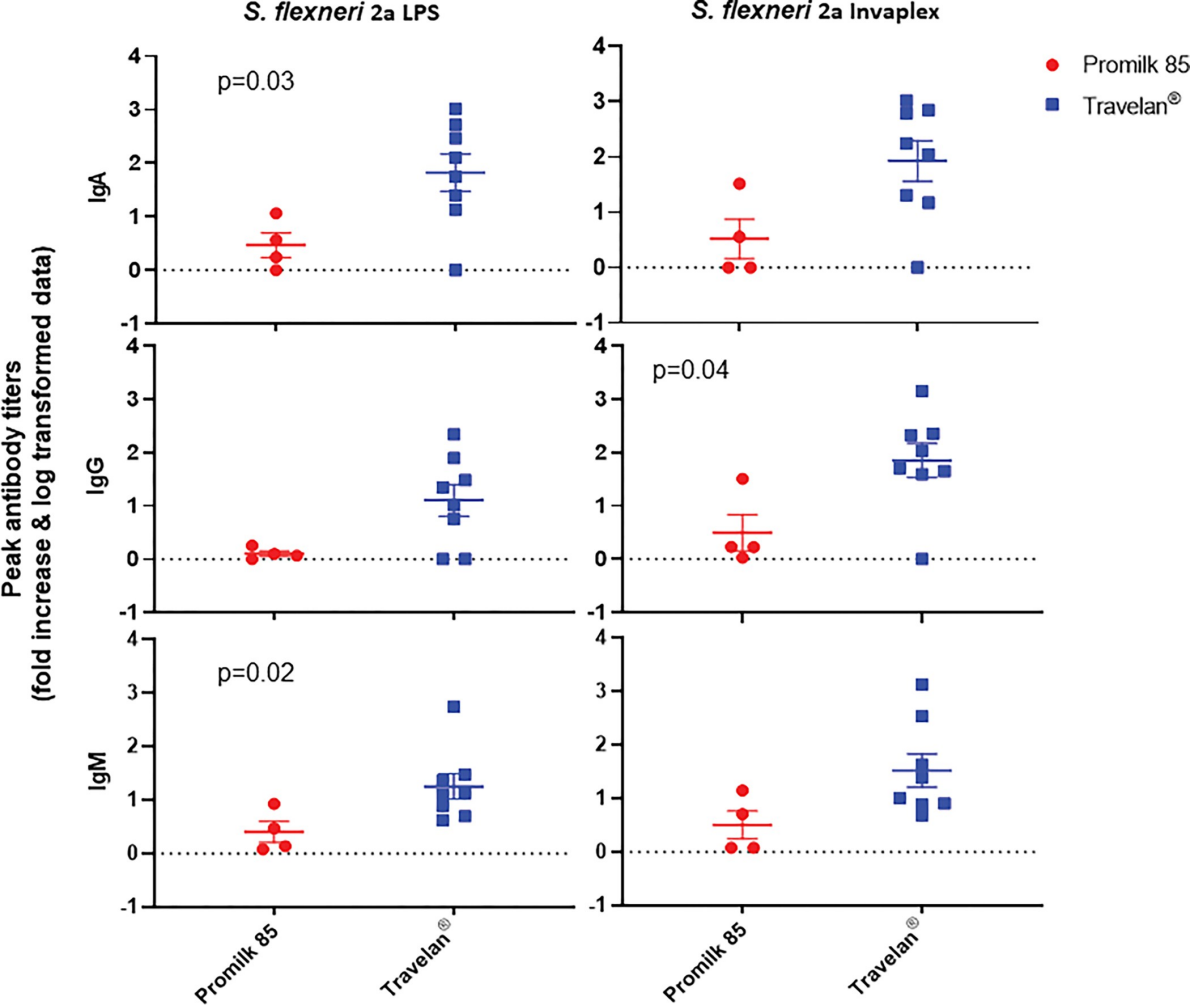
*Shigella*-specific IgA, IgG and IgM antibody responses following challenge. Serum IgA, IgG and IgM antibody titers against *S*. *flexneri* 2a 2457T strain LPS and *S*. *flexneri* 2a 2457T Invaplex (which contains IpaB, IpaC and IpaD proteins and LPS) antigens in all NJRM infected with *S*. *flexneri* 2a 2457T strain. Red dots (Promilk 85 group) and blue squares (Travelan^®^ group) represent individual animal peak antibody titer as fold increase over baseline values. Mean values are shown by a horizontal line and error bars represent SEM. Statistical analysis was performed using Mann Whitney U analysis where values p <0.05 were significant.

To assess the effects of Travelan^®^ on adaptive pathogen-specific immune response the IgA, IgG and IgM levels of antibodies in the serum of all NJRM was measured by ELISA. The last serum sample collected from animals in the Promilk 85 group was 3–5 days after challenge, within this time frame animals could not mount peak antibody response against *Shigella* antigens (Fig 7).

Only the 6 healthy Travelan^®^ treated animals that survived the entire study had a >4 fold rise in IgA, IgG and IgM antibody titers against both *S*. *flexneri* 2a LPS and Invaplex antigens compared to pre-challenge sera. This suggests that prophylactic treatment with Travelan^®^ prevents shigellosis and allows the development of immunity against this pathogen.

The elicited antibody responses between Travelan^®^ and Promilk 85 treated animals were compared. Whilst the trend was higher immune responses for the Travelan^®^ group, only the IgA (p = 0.03) and IgM (p = 0.02) antibody titers against *S*. *flexneri* 2a LPS and IgG (p = 0.04) antibody titers against Invaplex were significantly higher in the Travelan^®^ group (Fig 7).

## Discussion

Oral administration of immunoglobulin preparations from human serum as well as bovine colostrum and serum have been clinically evaluated and proven to be safe and effective against a variety of enteric microbial infections [18, 29–41]. Prophylactic administration of HBC, as well as HBC-derivatives, have had substantial benefits on childhood infectious diarrhea [33, 70–73]. Administration of HBC rich in pathogen-specific immunoglobulins were experimentally evaluated as a preventive or therapeutic modality for a variety of enteric infections, including ETEC [20, 27, 29, 33, 36, 39, 48]. Additionally, HBC has positive effects on the human immune system beyond binding to potential human-relevant pathogens. Bovine IgG binds to human Fc receptors which enhances phagocytosis, killing of bacteria and antigen presentation, and bovine IgG supports gastrointestinal barrier function demonstrated with *in vitro* models [46, 74].

In the absence of widely available and licensed vaccines for *Shigella*, ETEC or *Campylobacter*, alternative non-antibiotic immunoprophylactic treatments, such as Travelan^®^ hold tremendous value. As expected, Western blot analysis confirmed that antibodies in Travelan^®^ reacted to proteins in whole cell lysates from ETEC as well as whole cell lysates from *Shigella* and purified *Shigella* antigens. Travelan^®^ cross-reactivity with *Shigella* is not unexpected, as antibodies to cytosolic housekeeping proteins are conserved among *E*. *coli* and the closely related *Shigella* species. *Shigella* are highly genotypically similar to *E*. *coli*, and could be considered belonging to the same species [75]. The correlation between protective efficacy and the *in vitro* cross-reactivity of Travelan^®^ to cell lysates isolated from other enteric pathogens remains to be evaluated. Production of an effective multi-pathogen HBC product will likely require antigenic contribution from all target pathogens, rather than relying on antibody cross-reactivity induced after ETEC-only bovine immunizations.

Development of an effective HBC product may reduce the burden of multidrug resistant enteric bacteria. A search is underway to discover effective non-antibiotic drugs and small molecules to prevent diarrhea. For example, polypropyletherimine dendrimer glucosamine (PETIM-DG), an oral Toll Like Receptor-4 (TLR4) antagonist, can control resolution of the infection-related-inflammatory response and prevent neutrophil-mediated gut wall disruptions. HBC specific for *Shigella flexneri* can protect from *S*. *flexneri* challenge [36], and moreover they have shown that a higher titer of antibodies in HBC can better protect against necrosis often observed in severe shigellosis. However, other studies have demonstrated that HBC from cows immunized with *Shigella* did not have an effect on *Shigella* dysenteriae-infected children (1–12 years of age) in Bangladesh [76]. Combinations of different prophylactics modalities, such as combining HBC with probiotics, may also hold promise. A combination of HBC antibodies and a probiotic strain of *Lactobacillus* (or engineered derivatives) was found to be more effective than HBC alone in reducing diarrhea in a rotavirus mouse model [77]. Probiotics, pre-biotics, and passive immunoprophylactic have been identified as potential alternatives or products to be used in combination with HBC to increase efficacy.

In the current study, Travelan^®^ prophylaxis prevented clinical shigellosis (bacillary dysentery) in 75% of Travelan^®^ treated NJRM compared to a Promilk 85 placebo control treatment. These results clearly demonstrated that Travelan^®^ protected animals from shigellosis. This apparent neutralization of *Shigella* may have occurred by blocking bacterial attachment to the intestinal wall and preventing invasion, facilitating bactericidal activity, or other immune mechanisms. There was a significant increase of *Shigella*-specific antibody titers in the Travelan^®^-treated NJRM after oral infection with *S*. *flexneri* 2a, 2457T, even in the absence of severe disease. HBC treatment may protect from severe disease while facilitating antigen uptake and presentation to the immune system, allowing for generation of pathogen-specific immune responses, though further investigation is necessary to confirm. Activation of the adaptive immune system and generation of immune effectors such as antibodies and the induction of memory B cells in the absence of disease may play a substantial role in protection when HBC is no longer present in the gut. If such mechanisms are intact, HBC could provide protection from infection, still allowing the generation of “natural immunity” against enteric pathogens.

Understanding the molecular basis of the interaction between effectors of *Shigella* and host immune systems has advanced greatly during the past decade, which revealed the importance of manipulation of the host immune system during bacterial infection. *Shigella* delivers a subset of virulence proteins via the type III secretion system (T3SS) that enable bacterial evasion from host immune systems, allowing for efficient colonization of the intestinal epithelium. The T3SS harbored by *Shigella* is pivotal to infection, approximately 50 T3SS effectors of *Shigella* are currently recognized, and only one third of these have been elucidated at the molecular level [78]. *Shigella* secretes virulence factors that induce severe inflammation and mediate enterotoxic effects on the colon, producing the classic watery diarrhea seen early in infection. Once ingested, *S*. *flexneri* delivers shigella enterotoxin 1 (ShET1) in the jejunum to elicit fluid secretion [79]. *Shigella* also induces focally distributed ulceration in the intestine [80].

In the current study, NJRM treated with Travelan^®^ prophylaxis and challenged with a virulent of *Shigella* were largely protected from disease. While a subset (2/8) of Travelan^®^-treated NJRM did exhibit diarrhea symptoms, the disease was less severe compared to placebo Promilk 85 treated NJRM. Despite the extensive research, pathophysiology of shigellosis remains incompletely understood and correlates of protection against shigellosis have not been defined. The largest immune network in the body resides in the gut. Cytokines play a crucial role in driving, perpetuating, resolving, and wound healing of intestinal inflammation and inflammation is largely the body’s defense. The pro-inflammatory cytokine IL-6, and to a lesser degree IL-8, are involved in acute invasive gastroenteritis, such as shigellosis [81]. NJRM which succumbed to disease had a very high levels of IL-6 in fecal extract samples, indicating the IL-6 levels correlated with disease severity. Moreover, we have shown that inflammation in gut tissues is co-related to excreted inflammatory cytokines as well as *Shigella* colonization. A more complete understanding of interactions between the host microbiome and enteric pathogens and mechanisms by which HBC can reduce inflammation in the intestinal environment may supply important information to design a more effective prophylactic HBC product. The broadly conserved Ipa proteins (present in all *Shigella* serotypes) are attractive targets and perhaps should be included in future HBC formulations for evaluation in animal models of efficacy, which may provide coverage against all *Shigella* serotypes [82–85].

Collectively, the results of this study confirmed cross-reactivity of Travelan^**®**^ against *Shigella*, and showed 75% protection against shigellosis in NJRM model, however, additional refinement is required to increase product efficacy. Active research is underway to increase the antigenic breadth and quality of the antibodies included in the HBC product while evaluating approaches to effectively deliver the HBC to the GI tract. Success in these areas could translate into an effective immunoprophylactic for enteric disease prevention and therapy. This HBC product could prevent increasing antimicrobial resistance and could revolutionize current management of diarrhea caused by bacterial pathogens such as *Shigella*, *Campylobacter* and *Vibrio cholera spp*., yielding improved outcomes for patients and bolstering public health.
